# Role of Neuroinflammation in Amyotrophic Lateral Sclerosis: Cellular Mechanisms and Therapeutic Implications

**DOI:** 10.3389/fimmu.2017.01005

**Published:** 2017-08-21

**Authors:** Jia Liu, Fei Wang

**Affiliations:** ^1^Department of Neurology, Shengjing Hospital of China Medical University, Shenyang, China; ^2^Department of Orthopedics, Shengjing Hospital of China Medical University, Shenyang, China

**Keywords:** amyotrophic lateral sclerosis, neuroinflammation, microglia, astrocytes, neural stem cells, regulatory T cells, dl-3-*n*-butylphthalide

## Abstract

Amyotrophic lateral sclerosis (ALS) is a progressive neurodegenerative disease that affects upper motor neurons (MNs) comprising the corticospinal tract and lower MNs arising from the brain stem nuclei and ventral roots of the spinal cord, leading to fatal paralysis. Currently, there are no effective therapies for ALS. Increasing evidence indicates that neuroinflammation plays an important role in ALS pathogenesis. The neuroinflammation in ALS is characterized by infiltration of lymphocytes and macrophages, activation of microglia and reactive astrocytes, as well as the involvement of complement. In this review, we focus on the key cellular players of neuroinflammation during the pathogenesis of ALS by discussing not only their detrimental roles but also their immunomodulatory actions. We will summarize the pharmacological therapies for ALS that target neuroinflammation, as well as recent advances in the field of stem cell therapy aimed at modulating the inflammatory environment to preserve the remaining MNs in ALS patients and animal models of the disease.

## Introduction

Amyotrophic lateral sclerosis (ALS) is a fatal neurodegenerative disease characterized by progressive degeneration of upper and lower motor neurons (MNs) in the brain and spinal cord. ALS usually begins in limb or bulbar muscles, spreads to other body regions, and eventually ends with respiratory muscle dysfunction. The majority (more than 90%) of ALS is sporadic, whereas a minor fraction (about 5–10%) is familial. Mutations in Cu/Zn superoxide dismutase 1 (SOD1), the first identified gene in ALS, characterize more than 20% of familial and 1–4% of sporadic ALS cases (1, 2). To date, more than 20 gene mutations have been identified in familial ALS, and hexarepeat expansion on chromosome 9 open reading frame 72 (C9orf72) is reported to be the most frequent genetic cause of familial ALS (40%) (2, 3). Scientific advances in genetic studies have enabled the identification of genes contributing to ALS pathogenesis. However, no effective therapy is currently available for ALS patients. Riluzole, licensed by the Food and Drug Administration (FDA) in 1996, is the only drug that could extend the survival of ALS patients by about 3 months (4, 5). Therefore, it is urgent to identify new potential therapeutic target(s) for the development of more effective and beneficial treatments for ALS patients. In this review, we discuss not only the detrimental roles but also the immunomodulatory actions of key inflammatory cellular players in ALS. In addition, we summarize current pharmacological strategies targeting neuroinflammation and finally explore advances in stem cell therapy aimed at modulating the inflammatory environment to preserve the remaining MNs in ALS patients and animal models of the disease.

## Pathophysiology

Despite numerous preclinical and clinical studies that have been conducted to evaluate the underlying cause of MN degeneration, the exact pathogenic mechanism of ALS is still far from being understood. Several pathways, including oxidative stress, mitochondrial dysfunction, axonal damage, excitotoxicity, neuroinflammation, and protein aggregation, have been suggested to be involved in ALS pathogenesis (6). It seems that multiple factors, rather than a single mechanism, contribute to the development and progression of ALS.

Increased levels of free radicals were found in cerebrospinal fluid (CSF), serum, and urine samples of both familial and sporadic ALS patients (7–9). In fact, SOD1 plays a crucial role in the clearance of reactive oxygen species (ROS) and the aberrant activity of mutant human SOD1 (mSOD1) in ALS leads to oxidative damage (10). However, other ALS-related proteins, such as mutant TAR DNA-binding protein-43 (TDP-43) and other still unknown factors in sporadic ALS, may promote oxidative stress in MNs (11).

Accumulation of misfolded mSOD1 in mitochondria leads to morphological alternations such as vacuolated and dilated organelles with disorganized cristae and membranes in spinal MNs and skeletal muscles of both ALS patients and SOD1^G93A^ mice (12, 13), as well as physiological changes including abnormal release of adenosine triphosphate (ATP) and ROS, impaired energy homeostasis, and unusual activation of apoptosis (12–14). The presence of mSOD1 also contributes to altered mitochondrial calcium buffering capacity and reduced calcium uptake from the cytoplasm in SOD1^G93A^ mice (15, 16). In addition, axonal transport of mitochondria along microtubules is disrupted in ALS, leading to metabolic changes in neurons (12, 17).

Increased glutamate levels in CSF (18) and therapeutic benefits achieved by riluzole, an anti-excitotoxic drug (19), implicate excitotoxicity as a mechanism contributing to MN injury in ALS. Low endogenous calcium buffering capacity in ALS-vulnerable spinal and brainstem MNs makes them more susceptible to excitotoxic insults (20). It was reported that sera from ALS patients could induce abnormal *N*-methylaspartate receptor activation (21). Glutamate transport deficits have been identified in motor cortex and spinal cord of ALS patients and transgenic mSOD1 mouse models. Especially affected is astroglial-specific excitatory amino-acid transporter 2 (EAAT2) (22–24), leading to increased synaptic glutamate concentration and overstimulation of postsynaptic glutamate receptors, which contributes to excitotoxic neuronal degeneration (8, 25, 26). Guo et al. observed a delay in MN degeneration and disease progression in transgenic mSOD1 mice overexpressing EAAT2 (27), suggesting the loss of EAAT2 contributes to MN degeneration in ALS.

Aberrant mutant or misfolded proteins (e.g., SOD1, TDP-43, and FUS/TLS RNA-/DNA-binding protein) aggregate in the cytoplasm, nucleus, or extracellular matrix leading to cellular organellar damage and neuronal dysfunction in ALS (8, 28–32). Misfolded mSOD1 aggregates formed pore-like structures in lipid membrane that allowed the influx of calcium (33) and were even able to activate microglia *in vitro* (34). The ubiquitin proteasome system (UPS) and autophagy play a central role in degrading misfolded proteins and thus preventing their aggregation. Impairment of autophagy in MNs may result in the accumulation of misfolded proteins and cell death (35). Alternation of UPS and activation of autophagy have been observed in spinal MNs of mSOD1 mice (36) and in postmortem samples of ALS cases (37). The enhancement of autophagy could improve the clearance of misfolded protein aggregates and neuronal survival in ALS models (38, 39).

Analysis of CSF and postmortem spinal cord samples from ALS cases revealed increased microglial activation and lymphocyte permeation (40, 41), indicating that neuroinflammation may play a role in MN degeneration. Further investigation revealed that microglia were activated in the early stages of ALS and played an either deleterious or beneficial role (42, 43). Moreover, astrocytes acquired toxic properties and subsequently contributed to MN death (44), while infiltrated T lymphocytes controlled microglial response by limiting their detrimental effects and enhancing their neuroprotective capacity (45). In addition, the breakdown of blood–brain barrier and blood–spinal cord barrier also contributed to early MN degeneration in ALS patients and mice (46, 47), while restoration of the barrier integrity delayed the onset of neurodegeneration and disease progression (48).

## Neuroinflammation in ALS: Perspective on Cellular Basis

Neuroinflammation, characterized by microglial and astrocyte activation, T lymphocyte infiltration, and overproduction of inflammatory cytokines, has been demonstrated in association with neuronal loss in both animal and human tissues, even during the presymptomatic phase of ALS (49). Accumulating evidence from preclinical work has implicated immune cells in either exerting deleterious or protective effects on MN survival depending on the stage of disease progression; however, the mechanism is far from being fully elucidated.

### Microglia

Microglia are the first line of immune defense in the brain and spinal cord. They survey the surrounding environment and respond to “danger signals” from damaged tissues. It has been reported that injured MNs and astrocytes release misfolded proteins (such as mSOD1) in ALS, which activate microglia through CD14, toll-like receptor (TLR) 2, TLR4, and scavenger receptor dependent pathways (34, 50, 51). Direct evidence was provided using positron emission tomography (PET) that widespread microglial activation was present in the brain of living ALS patients and SOD1^G93A^ mice (52–54), with a significant correlation between the intensity of microglial activation in the motor cortex and the severity of clinical MN deficits (54). Studies in mSOD1 transgenic mice further revealed that the replacement of mSOD1 microglia with wild-type microglia, as well as reduced mSOD1 expression in microglia, postponed MN degeneration and extended the lifespan of the animals (55, 56). A recent work by O’Rourke et al. (57) demonstrated that C9orf72 expression was highest in myeloid cells, and the loss of C9orf72 function in mice led to defects in lysosomal trafficking, decreased ability of microglia to clear aggregated proteins, as well as altered microglial responses, and neuroinflammation. Similar results were observed in macrophages. In particular, even haploinsufficiency of C9orf72 appears to contribute to altered inflammatory responses in macrophages. These findings suggest that C9orf72 may have a dual effect on both neurons and myeloid cells.

In addition, extracellular ATP released by dying and abnormally functioning neurons may activate microglia through the ionotropic P2X and metabotropic P2Y purinergic receptors, followed by inflammatory reactions (58). The expression level of P2X_7_ was increased in activated microglia from postmortem spinal cord of ALS patients (59), as well as in SOD1^G93A^ mice (58). Furthermore, it was observed that the upregulation of P2X_4_, P2X_7_, and P2Y_6_ receptors in mSOD1 microglia, in particular P2X_7_, was associated with reduced ATP hydrolysis in the same ALS microglia, which led to increased production of tumor necrosis factor (TNF)-α and cyclooxygenase-2 (COX-2) with consequent toxicity to neuronal cells (60). This toxicity through activation of P2X_7_ was also confirmed in mSOD1 astrocytes (61). Studies in advance have shown that microglia-mediated deleterious effects in ALS could be prevented by genetic ablation of P2X_7_ receptor or by using specific antagonists to the receptor (58, 59, 62). However, further work displayed the complex role of P2X_7_ in ALS pathogenesis. Apolloni et al. found that constitutive deletion of P2X_7_ receptor aggravated disease progression, exacerbated astrogliosis, microgliosis, and motoneuron loss, activated MAPKs pathway, as well as increased the release of proinflammatory markers such as nicotinamide adenine dinucleotide phosphate oxidase 2 (NOX2) and inducible nitric oxide synthase in the lumbar spinal cord of end-stage (23 weeks of age) SOD1^G93A^ mice (63). Furthermore, these studies demonstrated that only the administration of P2X_7_ antagonist, Brilliant Blue G, starting at late presymptomatic stage (100 days/14 weeks of age) significantly enhanced MN survival in lumbar spinal cord through reducing microgliosis and modulating the expression of inflammatory markers, accompanied by delayed onset and improved motor functions (64). The key feature that emerged from these results might be the dual action of P2X_7_ and the existence of a time window concerning its beneficial role in ALS. The dual action of P2X_7_ during ALS progression might correspond to the switch of microglia from protective M2 to deleterious M1 phenotype. Therefore, precocious ablation of the receptor is detrimental, and its pharmacological blockade at early presymptomatic phase or after disease onset might be too early or too late. Thus, only interventions within an effective therapeutic window may provide positive outcomes in ALS patients.

Once activated, microglia display distinct and plastic phenotypes, with either neurotoxic or neuroprotective function, depending on the state of activation and disease stage. During the early slow progressive stage of ALS, microglia display an M2 phenotype with upregulated expression of M2 markers such as CD206 and Ym1, promote tissue repair and regeneration, and interact with protective signals such as CD200 and fractalkine (51, 65). As the disease progresses, injured MNs release “danger signals” (e.g., mSOD1) that induce microglia to acquire an M1 phenotype with enhanced secretion of NOX2, ROS, and proinflammatory cytokines (e.g., TNF-α, IL-1, and IL-6) (66, 67). *In vitro* co-culture studies further demonstrate that early stage M2 microglia enhanced MN survival, while end-stage M1 microglia were toxic to MNs (66).

### Astrocytes

Genes linked to ALS are not only expressed in MNs but also in astrocytes (68–71). Astrocytes expressing mSOD1 have been shown *in vitro* and *in vivo* to be toxic to both normal MNs and MNs derived from embryonic stem cell (ESC) carrying mSOD1 gene (71–73). Interestingly, mSOD1-expressing astrocytes selectively caused death of spinal MNs in ALS, but not spinal GABAergic or dorsal root ganglion neurons or ESC-derived interneurons (71). Selective silencing or blockage of mSOD1 gene in astrocytes, or transplantation of healthy astrocytes, could attenuate astrocyte-mediated toxicity and MN loss, delayed disease progression, and prolonged the lifespan of mSOD1 mice (74–77). Conversely, transplantation of mSOD1-expressing astrocytes induced focal MN degeneration and death in the spinal cord of wild-type rats (78). In addition, astrocytes reprogrammed from ALS patient fibroblasts impaired the survival of MNs (79, 80). Thus, the expression of ALS-associated mutant proteins in astrocytes contributes to non-cell autonomous toxicity. Qian et al. (79) recently showed that both MNs and non-MNs degenerated in the spinal cord transplanted with ALS astrocytes. Importantly, they observed that non-MNs were lost earlier than MNs, suggesting that non-cell autonomous toxicity by ALS astrocytes on neural degeneration is not specific to MNs, and non-MNs might mediate the degeneration.

As discussed above, astrocytes play an important role in ALS; however, it is still unclear how ALS-associated mutant proteins contribute to the dysfunction of astrocytes and how dysregulated astrocytes exert non-cell autonomous toxicity to MNs. In normal conditions, astrocytes clear excess glutamate from synaptic clefts through glutamate transporters. In sporadic and familial ALS patients as well as mSOD1 mice, the loss of glutamate transporter, EAAT2/GLT-1, led to less efficient uptake of glutamate by astrocytes and therefore exacerbated MN degeneration (22, 78, 81, 82). Mitochondrial defects in mSOD1 but not wild-type astrocytes were reported to be toxic for MNs (83), and this could be prevented by antioxidants and nitric oxide synthase inhibitors (83, 84). Ferraiuolo et al. revealed the metabolic dysfunction in ALS astrocytes, particularly in the astrocyte lactate efflux transporter, with resultant decrease in spinal cord lactate levels (85). In addition, astrocytes from postmortem tissues from ALS cases and SOD1^G93A^ mice were reported to exert toxic effects on MNs by secreting inflammatory mediators such as prostaglandin E2, leukotriene B4, nitric oxide, and NOX2 (76, 84, 86). Recently, astrocytes have been demonstrated to trigger MN death by activating a caspase-independent form of programmed cell death called necroptosis, which involves the loss of plasma membrane integrity through receptor interacting serine/threonine-protein kinase 1 (RIPK1) and mixed lineage kinase domain-like (MLKL) (87). Inhibition of the key necroptosis effectors, RIPK1 or MLKL, could protect MNs against sporadic ALS astroglial toxicity and delay the onset of motor dysfunction (87, 88), therefore suggesting these as potential new therapeutic targets.

### T Lymphocytes

In SOD1^G93A^ mice, CD4^+^ T cells were observed in lumbar spinal cords at early stages of the disease, while at the end stage, both CD4^+^ and CD8^+^ T cells were present (45, 89). Genetic removal of CD4^+^ T cells or functional total T cells accelerated disease progression with upregulated expression of NOX2 and proinflammatory cytokines in mSOD1 mice (90, 91), while reconstitution of T cells could prolong the survival of mSOD1 mice and inhibited the activation of M1 microglia (91). The neuroprotection associated with CD4^+^ T cells in ALS is probably due to their interactions with microglia and astrocytes. Beers et al. (92) reported that endogenous regulatory T cells (Tregs) were increased in spinal cords of mSOD1 mice at early slow progressive disease stage, accompanied by increased expression of IL-4 and levels of protective M2 microglia, while these decreased when disease rapidly accelerated with loss of forkhead box P3 (FoxP3) expression in Tregs. Passive transfer of Tregs obtained from donor mSOD1 mice during the stable stage without *ex vivo* activation extended the stable disease progression phage and lengthened survival of recipient mSOD1 mice (92). Accordingly, in ALS patients, the number of Tregs decreased in blood and spinal cord tissues accompanied by reduced expression of FoxP3, transforming growth factor-β, IL-4 and GATA binding protein 3 (Gata-3) at the rapidly progressive stage, and the number of Tregs was inversely correlated with the progression rates and severity (92, 93). A further analysis revealed that ALS patients with low FoxP3 levels showed more rapid progression, indicating that low FoxP3 expression might be predictive of future rapid disease progression (93). However, it is still unknown whether Tregs from ALS patients still keep their suppressive capabilities. Recently, Beers et al. (94) demonstrated that Tregs from both slowly and rapidly progressing ALS patients were dysfunctional and exhibited reduced suppressive capabilities on the proliferation of responder T lymphocytes compared to Tregs from healthy volunteers. Moreover, Treg suppressive deficiency correlated with disease burden and rates of disease progression. This study further illustrated that the loss of suppressive capabilities of Tregs from ALS patients was not permanent. Tregs regained their suppressive capabilities when removed from their environment and expanded *in vitro*, thus suggesting a potential novel therapeutic strategy for ALS patients.

Evidence has been provided that the expression of Th17 related cytokines (IL-17 and IL-23) was elevated in blood, CSF, and spinal cord tissues from ALS patients, suggesting Th17 might also play a role in ALS pathogenesis (95–97). However, little is known about Th17 in mSOD1 transgenic mice. Another T-cell subpopulation participating in ALS is natural killer T (NKT) cells. Rentzos et al. reported that NKT cells were increased in peripheral blood of ALS patients (98). Moreover, NKT cell levels and activation state increased in the spinal cord, spleen, and liver of mSOD1 mice, and immunomodulation of NKT cells led to the reduction of MN loss, delayed disease onset, and prolonged the life span of mSOD1 mice (99).

### Monocytes/Macrophages

Ample previous evidence has uncovered the role of activated microglia in the CNS and altered T cells in the peripheral nervous system of ALS patients and mSOD1 mice as discussed above. However, much less is known about the role of peripheral macrophages and monocytes in MN degeneration. Several studies have reported the activation of monocytes in the peripheral blood of ALS patients (100), as well as the increased invasion of peripheral monocytes into the spinal cord of ALS patients and mice (101, 102), which contributed to MN loss. Activated monocytes from ALS patients exhibited decelerated phagocytosis, altered adhesion behavior, and impaired proinflammatory cytokine secretion (102, 103). A recent study revealed 233 differentially expressed genes in ALS monocytes. Among these was a unique inflammation-related gene expression profile, including IL-1β, IL-8, FOSB, CXCL1, and CXCL2, suggesting ALS monocytes were skewed toward a proinflammatory state in the peripheral circulation, which might play a role in rapidly progressing ALS (104). Murdock et al. further suggested that an increased ratio of neutrophils to monocytes displayed a better correlation with disease progression (105). Another recent study has observed macrophage-mediated inflammation in the skeletal muscle of familial ALS rats (106), which might be another therapeutic target for novel treatment of ALS.

### Complement System

The complement system plays a crucial role in the recruitment of mononuclear cells and macrophages (mediated by C3a and C5a) and in the deposition of cytotoxic pore-forming membrane attack complex (formed by C5–C9) on the cell surface. Multiple complement components including C1q, C5a, and C5b-9 are elevated in the plasma, CSF, and spinal cord of ALS patients and transgenic mSOD1 mice (107–110). However, the role of the complement pathway in ALS pathogenesis is still controversial. Complement factor C1q was observed to colocalize with neurofilament and deposited on motor end plates in intercostal muscle of ALS patients, suggesting that complement activation may precede end-plate denervation in human ALS (111). Lobsiger et al. showed that genetic deletion of C1q and C3 from ALS mice did not affect the overall onset and progression of the disease, thus suggesting that C1q induction and classical or alternative complement pathway activation do not contribute significantly to mSOD1-mediated ALS pathogenesis in mice (112). On the contrary, others have reported that terminal complement activation and C5a production occurred in skeletal muscle tissue of SOD1^G93A^ mice (113). Local activation and increased expression of C5a–C5aR1 signaling contributed to the recruitment of macrophages that might have accelerated muscle denervation and MN death in SOD1^G93A^ mice (113, 114). Selective inhibition of C5a–C5aR1 signaling ameliorated disease pathology, reduced motor symptoms, and extended the survival of SOD1^G93A^ mice (110, 115).

## Pharmacological Strategies Targeting Neuroinflammation in ALS

Multiple preclinical studies and clinical trials have been conducted to search for the underlying cause of MN degeneration; however, the exact mechanism remains largely unclear. Therefore, the development of effective and targeted therapies is still underway worldwide. In recent years, increased interest has been focused on finding appropriate tools to effectively target neuroinflammation in ALS. Multiple compounds with anti-inflammatory properties have been reported to enhance MN survival in transgenic mice, even though none has been shown to be effective for ALS patients (116). A number of neuroprotective agents targeting neuroinflammatory pathways in ALS are summarized in Table 1.

**Table 1:** Summary of drugs and compounds targeting neuroinflammation in ALS.

Drug	Mechanism	Trial no.	Phase	Status	Reference
Minocycline	Suppressing microglial activation and modulating apoptosis	NCT00047723	III	Failed	Gordon et al. (119)
NP001	Modulation of monocyte activation and downregulation of NF-kB in macrophages	NCT01281631 NCT02794857	II	Completed Ongoing	Miller et al. (120)
Masitinib	Tyrosine-kinase inhibitor	NCT02588677	II and III	Completed	Trias et al. (122)
Ibudilast (MN-166)	Phosphodiesterase 4 inhibitor	NCT02238626 NCT02714036	II	Ongoing Ongoing	Martinez et al. (123)
Fingolimod	Sphingosine-1-phosphate receptor modulator	NCT01786174	II	Completed	Potenza et al. (125)
RNS60	Modulator of PI3K–Akt pathway	NCT02525471	I	Active, not recruiting	Crisafulli et al. (126)
Tocilizumab	IL-6 receptor antagonist	NCT02469896	II	Ongoing	Mizwicki et al. (130)
Anakinra	IL-1 receptor antagonist	NCT01277315	II	Not known	Maier et al. (129)
Celebrexib	COX-2 inhibitor	NCT00355576	II	Failed	Cudkowicz et al. (133)
Thalidomide	TNF-α antagonist	NCT00140452	II	Failed	Stommel et al. (131)
Lenalidomide	TNF-α antagonist	N/A	N/A	N/A	Neymotin et al. (132)
AMD3100	CXCR4 antagonist	N/A	N/A	N/A	Rabinovich-Nikitin et al. (128)
NBP	Anti-oxidant, reduction of glial activation	ChiCTR-IPR-15007365	II and III	Ongoing	Feng et al. (137)
Celastrol	Inhibition of inflammatory cytokines and induction of heat shock protein response	N/A	N/A	N/A	Kiaei et al. (134)

*ALS, amyotrophic lateral sclerosis; IL, interleukin; COX-2, cyclooxygenase-2; TNF, tumor necrosis factor; NBP, dl-3-n-butylphthalide; N/A, not available*.

Minocycline, a broad-spectrum tetracycline antibiotic, was shown to reduce the loss of MNs, delay disease onset, and extend survival of SOD1^G93A^ mice (117), probably by suppressing microglial activation and modulating apoptosis (118). Unfortunately, a phase III trial (ClinicalTrials.gov Identifier: NCT00047723) revealed harmful effects after its continuous administration to ALS patients (119), limiting its further application. NP001 is a small molecule that regulates monocytes and macrophages by switching them from an inflammatory phenotype to a basal non-inflammatory phenotype. In the completed phase I and II trials in ALS patients (ClinicalTrials.gov Identifier: NCT01091142, NCT01281631), NP001 was found to be safe, well tolerated, and presented a positive trend in slowing disease progression (120, 121). Currently, a second phase II trial is ongoing to confirm these data (ClinicalTrials.gov Identifier: NCT02794857). Masitinib is a tyrosine-kinase inhibitor that was observed to decrease aberrant glial cells, microgliosis, and MN degeneration in the spinal cord of mSOD1 mice, and therefore prolonged survival of the animals (122). A phase II and III clinical trial aimed at assessing the efficacy and safety of masitinib in combination with riluzole in the treatment of ALS patients (ClinicalTrials.gov Identifier: NCT02588677) has recently completed with a total of 394 patients enrolled, and the final results are expected to be announced soon. Ibudilast (MN-166), a non-selective phosphodiesterase 4 inhibitor, is reported to modulate the production of proinflammatory agents from resident immune cells, and also influences their survival and activation (123). Two clinical trials with ibudilast are ongoing in ALS patients. One is administration of ibudilast in combination with riluzole to evaluate its general safety and tolerability (ClinicalTrials.gov Identifier: NCT02238626). Another trial is to investigate the impact of ibudilast on neuroinflammation measured by PET imaging and blood biomarkers (ClinicalTrials.gov Identifier: NCT02714036). Fingolimod, a modulator of sphingosine-1-phosphate receptor, is the first oral drug approved by FDA for the treatment of relapsing remitting multiple sclerosis, because it reduces the number of circulating lymphocytes in peripheral blood by sequestering them in secondary lymphoid organs through its modulation of the sphingosine-1-phosphate receptor (124). In a recent preclinical study, fingolimod improved the outcome and survival rate of mSOD1 mice, and the beneficial effect was associated with modulation of microglial activation and innate immunity (125). A phase II trial evaluating the safety and tolerability of fingolimod in ALS patients (ClinicalTrials.gov Identifier: NCT01786174) has recently been completed with no major safety issue reported.

Blockage of proinflammatory mediators (e.g., IL-6, IL-1, TNF-α, COX-2, and CXCR4) in ALS to modulate neuroinflammation and decrease MN death is another strategy that resulted in delayed symptom onset and prolonged survival of mSOD1 mice, and clinical trials for these compounds are ongoing (4, 123, 126–134) (Table 1). dl-3-*n*-Butylphthalide (NBP) was initially isolated from the seeds of *Apium graveolens* (celery) and has been approved by the State FDA of China for clinical use since 2002. NBP has been demonstrated to exert neuroprotective roles in cerebral ischemia and vascular dementia (135, 136). In preclinical work, NBP reduced glial cell activation, attenuated MN death, and thus prolonged the survival interval of SOD1^G93A^ mice (137–139), suggesting this compound might be a novel treatment for ALS. A phase II and III multicenter, randomized, double-blind, placebo-controlled clinical trial with oral administration of NBP in ALS patients is ongoing in China to evaluate its efficacy and safety (Chictr.org.cn Identifier: ChiCTR-IPR-15007365).

## Cell Therapy for ALS

Stem cell therapy offers a promising alternative for ALS, and preclinical studies have demonstrated that cell-based treatment could increase MN survival and delay disease progression. Different sources and types of cells have been and/or are being tested in preclinical stages and clinical trials for ALS (140, 141).

Mesenchymal stem cells (MSCs) derived from adipose or bone marrow tissue are the most studied cells in ALS animals and clinical trials. Studies with MSCs grafted in mSOD1 mice have reported beneficial effects on disease progression including attenuated neuroinflammation, improved motor function, reduced loss of MNs, and prolonged survival (142, 143). MSCs may also serve as a carrier to deliver neurotrophic factors (144). Due to the positive results obtained from *in vivo* models of the disease, numerous cell-based clinical trials have used MSCs and reported the feasibility, safety, and immunomodulatory effects of administration of MSCs to ALS patients (142, 145, 146). However, adverse effects of MSCs, such as enhancing tumor growth and metastases (147, 148), and triggering ROS generation and inflammation (149), have also been reported. Thus, more careful and detailed studies concerning the clinical safety and efficacy of MSCs should be further performed.

Embryonic stem cells have the potential to differentiate into any cell types of the three germ layers, including specific cells of neuronal or glial fates. However, whether MNs derived from ESCs could exert beneficial effects on ALS animals is still controversial (150, 151). The application of ESCs is also limited due to the difficulty of generating high-purity lineage-specific cell lines without risk of tumorigenesis and ethical issues. The generation of induced pluripotent stem cells (iPSCs) provides an alternative therapeutic approach to cell-based treatment for ALS. Autologous iPSCs might represent an ideal cell source that can be derived from patients’ own somatic cells with similar features as ESCs, but with no ethical issues, reduced risk of immune rejection, and less need for immunosuppressive drugs. Transplantation of iPSC-derived neural cells into SOD1^G93A^ mice showed survival of donor cells, axonal sprouting, and reduction of macro- and microgliosis (152, 153). However, the use of iPSCs in the clinic is still under debate due to its capacity of tumor formation after transplantation, inefficient *in vitro* differentiation methods, and inherent genetic deficits from donors. Thus, it is still a big challenge to produce safe iPSC-derived neural cells for clinical cell therapy.

Neural stem/progenitor cells (NPCs) are multipotent cells committed to the neural cell lineage that can self-renew and be readily expanded *in vitro*. NPCs derived from an already formed nervous system have not been reported to cause tumor formation with metastasis after transplantation, which is an important criterion for a donor cell population in clinical cell transplantation. However, the limited replication and decreased differentiation potential with time, as well as the ethical concerns imposed by NPC origins and derivation should not be ignored. NPCs, derived from adult and fetal CNS tissues, as well as from ESCs and iPSCs as mentioned above, have been reported to survive, differentiate into mature neural cells, and may delay disease progression in association with extended lifespan after transplantation in ALS animals (154). The potential mechanism of grafted NPCs to repair injured MNs has been widely investigated. It has been demonstrated that NPCs may provide an effective treatment by directly replacing the lost and damaged neural cells, enhancing functional synaptic formation, providing neuroprotection by producing neurotrophic factors, or even stimulating endogenous neurogenesis (153, 155–158). As discussed previously, neuroinflammation is a key player in ALS pathogenesis that contributes to MN degeneration. However, it is still unclear whether grafted NPCs are able to modulate the inflammatory environment around damaged MNs in ALS and how donor NPCs interact with host immune cells. NPCs were reported to inhibit T-cell proliferation and promote apoptosis of encephalitogenic CNS-infiltrating T cells through apoptotic receptor ligands (e.g., FasL and Apo3L) and release of soluble mediators (e.g., nitric oxide synthase, leukemia inhibitor factor, heme oxygenase-1, and prostaglandin E2) in the chronic inflammatory environment of experimental autoimmune encephalitis (159–164). In spinal cord injury, grafted mouse NPCs could lead to a reprogramming of the local inflammatory microenvironment from a “hostile” to an “instructive” state by increasing the proportion of Tregs and reducing that of M1 macrophages (165). In our previous work with *in vitro* allogeneic co-culture settings, we demonstrated that human fetal-derived NPCs reduced proliferation of peripheral blood mononuclear cells *via* the upregulation of Tregs (166) and influenced allogeneic microglial activation and functional activity through enhanced CD200-CD200R interaction between NPCs and microglia (167). A recent work by Gao et al. indicated that induced NPCs directly reprogrammed from mouse embryonic fibroblasts were able to influence microglia activation and the acquisition of neuroprotective phenotypes *via* CXCL12/CXCR4 signaling (168). Further studies concerning the immunomodulatory effects of grafted NPCs in mSOD1 rodents and even ALS patients are urgently needed. Due to the encouraging results of preclinical studies with neural cell therapy, several clinical trials with human NPCs or human glial restricted progenitor cells are either completed or ongoing (Table 2). Till now, the donor cells have been well tolerated and no safety issue has been reported (169–175).

**Table 2:** Clinical trials of human NPCs in ALS.

Cell type	Donor cells	Delivery method	Adjutant treatment	Follow-up (months)	Trial no.	Phase	Status	Reference
SpC-NPC	NSI-566RSC	Intraspinal	Basiliximab	18	NCT01348451	I	Completed	Glass et al. (174)
			Tacrolimus					Riley et al. (173)
			Mycophenolate					Riley et al. (172)
								Feldman et al. (171)
								Tadesse et al. (175)

SpC-NPC	NSI-566RSC	Intraspinal	Basiliximab	24	NCT01730716	II	Completed	Glass et al. (169)
			Tacrolimus					
			Mycophenolate					

NPC	Fetal NPCs	Intraspinal	Tacrolimus	18	NCT01640067	I	Completed	Mazzini et al. (170)

NPC	CNS10-NPC-GDNF	Intraspinal	N/A	N/A	NCT02943850	I/IIa	Ongoing	ClinicalTrials.gov

GRP	Q-Cells	Intraspinal	N/A	9	NCT02478450	I/IIa	Active, not recruiting	ClinicalTrials.gov

*ALS, amyotrophic lateral sclerosis; NPC, neural stem/progenitor cell; SpC-NPC, human spinal cord-derived NPC; GRP, human glial restricted progenitor cell; N/A, not available*.

## Conclusion

Effective therapy for ALS is still in its infancy, even after years of intense investigation with chemical compounds and cell-based treatment. The pathogenesis involved in MN death in ALS is complex, and neuroinflammation has been accepted as a key contributor to MN degeneration and disease progression. However, a big challenge regarding the development of new therapies for ALS patients is the failure to translate positive preclinical results into successful clinical practice. Several issues should be taken into consideration when designing therapeutic strategies targeting neuroinflammation in ALS. First, most preclinical ALS studies invariably employ the mSOD1 transgenic rodents (generally SOD1^G93A^ mice), which is a limited disease condition related to a small subgroup of ALS patients with SOD1 gene mutation. Therefore, transgenic mouse models with different gene mutations (e.g., TDP-43 and C9orf72) should be employed in preclinical studies to find out whether they share common mechanisms involved in neuroinflammation. Second, therapeutic strategies in transgenic animals are usually applied during presymptomatic or slowly progressive disease stage. Despite promising treatment outcomes in these preclinical studies, they cannot be translated into patients because most ALS patients are identified and diagnosed during the late and rapidly progressive phase. Third, the inflammatory environment of ALS varies with disease progression, and involves both neurotoxic and neuroprotective aspects. Thus, specific therapeutic timing may influence the pathogenic target and choice of drugs. Fourth, the heterogeneity of patients may also contribute to the failed translation of therapeutic effects from homogeneous transgenic animals to ALS patients. In addition, one should consider promoting anti-inflammatory and neuroprotective properties of immune cells, instead of simply and completely suppressing inflammatory and immune responses to achieve precise and personalized treatment for ALS patients.

## Author Contributions

JL and FW wrote the manuscript and approved the final manuscript.

## Conflict of Interest Statement

The authors declare that the research was conducted in the absence of any commercial or financial relationships that could be construed as a potential conflict of interest.
